# Health Care Professionals’ Perspectives on Technology Use in Urinary Care: Cross-Sectional Survey-Based Study

**DOI:** 10.2196/73453

**Published:** 2025-07-24

**Authors:** Nicole Zhang, Ni Zhang, Yun Wang, Rachel Wong, Kaylin Ma

**Affiliations:** 1 Valley Foundation School of Nursing College of Health and Human Sciences San Jose State University San Jose, CA United States; 2 Department of Public Health and Recreation College of Health and Human Sciences San Jose State University San Jose, CA United States; 3 Biomedical Engineering Department Charles W Davidson College of Engineering San Jose State University San Jose, CA United States

**Keywords:** internet research, survey study, urology, technology, urinary health technology

## Abstract

**Background:**

Urinary issues pose a significant burden on health care systems, necessitating innovative solutions to enhance patient care and alleviate the provider burden.

**Objective:**

The objective of this study was to explore health care professionals’ perceptions of urinary issues and assess their acceptance and readiness to adopt wearable and remote technologies for managing these conditions. The study aimed to identify the attributes and barriers influencing technology integration in clinical practice, using established theoretical frameworks, such as the Health Belief Model (HBM) and the Technology Acceptance Model (TAM).

**Methods:**

A cross-sectional survey-based study was conducted. A structured survey questionnaire was administered online to a sample of 256 health care professionals recruited through social media and personal networks. The survey included both closed- and open-ended questions to gather data. Quantitative data were analyzed using descriptive statistics, Pearson correlation, and multiple regression.

**Results:**

Quantitative analysis revealed strong correlations between belief agreement and factors such as health literacy (*r*=0.591, *P*<.001), the perceived burden (*r*=0.628, *P*<.001), device attributes (*r*=0.650, *P*<.001), and support services (*r*=0.622, *P*<.001). Multiple regression analysis identified that the perceived burden (β=.284, *P*=.01), device attributes (β=.371, *P*<.001), and integrating technology (β=.312, *P*<.001) are positively associated. The survey demonstrated strong internal consistency, with Cronbach α=.85, indicating high reliability in measuring health care professionals’ perceptions of technology adoption.

**Conclusions:**

Health care professionals’ acceptance of technology in managing urinary issues is influenced by factors such as the perceived burden, device attributes, and the ease of integrating technology into existing workflows. Addressing barriers to technology adoption, providing comprehensive training and support, and prioritizing user-centered design are crucial for successful technology integration. Future research should focus on longitudinal studies and explore the perspectives of patients and other stakeholders to gain a more holistic understanding of technology integration in urological care.

## Introduction

### Background

Urinary issues are pervasive in health care across multiple settings. Common urinary issues encountered by health care professionals include conditions such as urinary tract infection (UTI), urinary incontinence (UI), benign prostatic hyperplasia (BPH), and urinary retention (UR). The prevalence of these conditions is staggering. As of 2021, there were an estimated 4.49 billion cases of UTIs worldwide, representing a 66.45% increase since 1990 [1]. The age-standardized incidence rate (ASIR) was 5531.88 per 100,000 population, with women and older adult men experiencing the highest incidence. Approximately 20 million American women and 6 million American men experience UI at some point in their lives, with prevalence increasing with age. In women over 30 years old, stress incontinence affects 24%-45%. The significance of addressing urinary issues is multifaceted. Clinically, UTIs are the second-most common type of infection in older adults, potentially leading to serious complications, such as kidney failure and even death if left untreated. These conditions impose a heavy burden on patients, as well as caregivers, influencing workflow management in health care settings. Economically, the impact is substantial [2].

Despite the prevalence of urologic conditions, these conditions often remain underdiagnosed and undertreated, especially among older adults and marginalized populations. Timely detection and effective management are essential but are frequently hindered by the limitations in current monitoring methods. These current methods rely heavily on self-reporting and episodic clinical assessments. Advances in health technology, particularly wearable and remote devices, offer promising solutions to bridge these gaps and enable real-time noninvasive monitoring of urinary symptoms and behaviors. Understanding health care professionals’ acceptance of these technologies is critical to their successful implementation in adoption and practice.

Although general technology integration in health care has received substantial attention, specific challenges in urinary care, such as underreporting, delayed diagnosis, and lack of real-time monitoring, remain under addressed. Urinary conditions, such as UI and lower urinary tract symptoms (LUTS), significantly affect the quality of life, particularly among older adults, yet they are often overlooked due to stigma and limited access to effective assessment tools [3]. Traditional care models rely heavily on self-reporting or episodic clinical assessments, which may fail to capture the dynamic nature of symptoms. Delay in identification and intervention can exacerbate morbidity and contribute to poor outcomes [4]. Emerging wearable and digital technologies offer a potential solution, enabling noninvasive, continuous monitoring to support earlier recognition and personalized interventions [5,6]. However, as of now, the adoption of these tools in urinary care remains limited. This study sought to explore health care professionals’ readiness to integrate such technologies, with particular attention to the barriers and enablers specific to urinary care delivery.

The staggering prevalence of urinary issues underscores the critical need for innovative solutions in health care. As we find ourselves in an era of rapid technological advancement, the integration of technology into medical practice has become not just beneficial but also necessary, especially when confronting such widespread health concerns.

Technology integration in health care has emerged as a key driver in improving patient care and operational efficiency. With the increasing complexity of health care delivery, stand-alone systems are no longer sufficient to address the multifaceted challenges posed by conditions such as UTIs and UI. The integration of various health care technologies offers a holistic approach to patient care, enabling seamless communication between different systems and departments. In the context of urinary health, technological advancements have led to the development of innovative tools and approaches. By leveraging technology integration, health care professionals can streamline operations, reduce administrative burdens, and focus more on delivering high-quality patient care. This approach is essential in tackling the growing prevalence of urinary conditions and improving overall health care efficiency. Although this is the case, there are many aspects to consider regarding health care professionals’ acceptability of technology in practice.

### Factors Surrounding Technology Acceptance in Health Care Settings

The integration of digital technologies in health care, ranging from electronic health records (EHRs) to artificial intelligence (AI) systems, promises to enhance patient care, safety, and clinical efficiency. However, successful implementation depends heavily on the acceptance of these technologies by health care professionals, including nurses, physicians, and allied staff. Understanding the factors influencing their acceptance is critical for designing effective interventions and implementation strategies.

#### Individual-Level Factors

Health care professionals’ willingness to adopt new technologies is often shaped by their individual traits. Personal innovativeness, self-efficacy, and readiness for change are strong predictors of technology acceptance. Studies have found that health care professionals with greater self-confidence in using digital tools are more inclined to adopt systems such as EHRs and telemedicine [7,8]. Conversely, psychological barriers, such as technophobia, resistance to change, and fear of reduced autonomy, can significantly hinder acceptance [9]. Addressing these internal barriers through education and peer support is a recurrent recommendation.

The perceived benefit of a system plays a pivotal role. If health care professionals believe a specific technology improves clinical workflow or patient outcomes, they are more likely to accept it [10,11]. This finding echoes across multiple studies, highlighting that practical, outcome-oriented value is central to user adoption.

#### Technology-Related Factors

A consistent theme in the literature is that ease of use and a user-friendly design facilitate acceptance. Technologies perceived as intuitive and minimally disruptive have higher adoption rates [10,12]. These characteristics align with the core components of the Technology Acceptance Model (TAM), which is frequently used as a guiding framework in the studies reviewed. System failures, bugs, and unreliability are significant deterrents. For instance, a study found that technical issues, especially with AI-based decision support tools, causes distrust and disengagement. Alert fatigue, particularly in decision support systems, is cited as a recurring concern among clinicians [9].

#### Organizational Context

Adequate infrastructure is a foundational requirement. Without access to a stable internet connection, compatible hardware, and technical support, even the most promising technologies face poor uptake [13]. Targeted and ongoing training programs are crucial to equip users with the skills needed to operate new systems. Studies consistently recommend that training be context specific, iterative, and responsive to user needs [9,11]. The involvement of clinical staff in the design and rollout of technology enhances buy-in. Implementation strategies that include stakeholder engagement, local champions, and organizational incentives have been found to increase adoption success [13].

#### Professional Practice Impact

Technologies that align well with clinical workflows are more readily accepted. Systems that disrupt routines or increase the burden face greater resistance [11,13]. Effective technologies must therefore be designed with a deep understanding of clinical operations. Many health care professionals express concern that technology, especially AI and decision support systems, may diminish their clinical judgment or autonomy [9]. This is less of a concern with more administrative technologies, such as EHRs, but remains relevant across the digital health spectrum. Perceived effects on patient care, either positive (eg, efficiency, safety) or negative (eg, depersonalization, error risk), strongly influence acceptance. Health care professionals are more open to using technologies they believe enhance patient outcomes [14].

### The Gap and How Technology Can Help

Technology offers promising solutions for managing urinary issues more effectively. Electronic alerts in EHRs have been shown to significantly reduce catheter-associated urinary tract infections (CAUTIs). Researchers have developed mobile uroflowmetry technology and urination log apps that use AI and acoustic analysis to assist in diagnosing urinary diseases [15]. Wearable sensing devices and Internet of Things (IoT) technologies can collect and analyze urination information for personal health care services and disease management. Overall, the use of easy-to-operate sensing technology is currently underused in addressing these common issues. Research in other domains, such as diabetes and cardiovascular care, has shown that health care professionals’ acceptance of wearable technology hinges on usability, integration into workflows, and institutional support [16,17]. These lessons provide a valuable context for understanding how similar factors may influence wearable technology adoption in urinary care.

Although there is a growing body of literature on wearable health technologies, most studies emphasize clinical efficacy or patient-centered outcomes. Few focus on health care providers’ acceptance, particularly for urinary applications. This study fills that gap by examining how nurses, physicians, and allied health staff perceive wearable and remote technologies for managing urinary issues, including their readiness to adopt such innovations into practice.

Our study addressed wearable and remote sensing technologies for urinary issues; however, we also addressed general health care technology exposure and acceptability. Specifically, in addition to urinary-specific technologies, the study also examined respondents’ general exposure to digital health tools, such as remote monitoring apps, wearables, and telemedicine platforms, which could influence their openness to adopting similar tools for urinary care. This approach allowed us to gain a comprehensive understanding of health care providers’ perceptions and readiness to adopt new technologies in their practice and the attributes of those technologies. The survey explored health care providers’ exposure to various technologies, both specific to urinary care and in general health care settings. This helped us assess their familiarity with different technological solutions and how this exposure might influence their acceptance of new technologies.

Previous research has found that technology is more likely to be accepted if there are fewer financial constraints, appropriate training, proper integration into current systems, and adequate technical support [18]. However, barriers to technology adoption include cost, efficient implementation and integration, and effective training. Broader considerations, such as resistance to change and lack of workflow integration, can also impact the efficiency of work and technology adoption.

### Addressing Gaps and Research Goals

This study aimed to fill gaps in previous research by investigating health care providers’ perceptions of urinary issues and exploring the use of technology for managing these issues. The research questions are as follows:

- What is the level of acceptance and readiness among health care providers toward adopting health care technology for treating and managing urologic issues?
- What attributes and barriers influence the clinical integration and utility of health care technologies for urinary issue management?

By addressing these research questions through a cross-sectional survey, the study sought to understand the major barriers and facilitators identified by health care professionals across multiple health care settings. This understanding will help facilitate the adoption of new technology, ultimately improving patient care and reducing the burden of urinary issues on health care systems. Health care professionals are the gatekeepers of technology implementation in clinical settings. Their acceptance is essential for technology to be successfully integrated into workflows, adopted by patients, and sustained over time.

The research used a comprehensive survey designed to capture health care professionals’ views across multiple health care settings. The survey was developed through a rigorous process, beginning with an extensive literature review to identify key themes and gaps in current knowledge. This was followed by consultations with subject matter experts in urology, nursing, and health technology to ensure the survey’s content validity.

### Theoretical Underpinnings

In this study, we used TAM as a guiding framework and integrated constructs from the Health Belief Model (HBM) to better understand health care providers’ attitudes, acceptance, and readiness toward adopting technologies for managing urologic issues. Both models provided complementary perspectives for examining behavioral drivers and perceived barriers to clinical technology integration.

TAM focuses on 2 primary constructs, perceived usefulness and perceived ease of use, as key predictors of technology adoption. These concepts aligned well with our exploration of how health care providers perceive and use technology in the management of urologic conditions. For example, their willingness to use health care technologies is often influenced by whether they believe these tools will improve efficiency, clinical outcomes, or patient care (perceived usefulness) and whether they feel the technology is easy to learn and integrate into existing workflows (perceived ease of use). These elements directly inform attitudes toward use and behavioral intentions, which were central to our investigation of acceptance and readiness.

To further deepen our analysis, we incorporated elements from the HBM, which emphasizes individuals’ perceptions of susceptibility, severity, benefits, and barriers in relation to health behavior. Applying the HBM allowed us to explore how health care providers’ beliefs about the importance of managing urologic issues may shape their motivation to adopt technology. For example, if a health care provider perceives urinary issues as a significant and underaddressed problem in clinical practice, they may be more motivated to adopt technologies they believe could mitigate these risks, especially if they believe such tools are beneficial and not overly burdensome. Generally, the HBM is helpful for framing beliefs around addressing health issues. This includes the modalities for addressing health issues, so this framework is helpful for assessing the facilitators of and barriers to technology adoption.

Items derived from TAM were evaluated as essential determinants of technology adoption, including perceived usefulness and perceived ease of use. These constructs are widely validated in studies examining the acceptance of health care technologies and are relevant for assessing health care professionals’ readiness to engage in technology usage.

To complement the behavioral focus of TAM, key constructs from the HBM, including perceived susceptibility, perceived severity, perceived benefits, and perceived barriers, were included to assess health-related motivations and the perceived consequences of adopting or resisting technology.

By combining TAM and the HBM, we were able to capture both the technological factors that affect adoption (eg, usability and perceived value) and the health-related beliefs that drive motivation and behavior in clinical decision-making. This dual-framework approach offered a robust structure for evaluating the multifaceted nature of technology integration in urinary care. Our survey instrument was developed using this dual-framework approach and included additional items drawn from validated questionnaires These theories provided complementary perspectives on technology adoption in health care settings, offering a comprehensive framework for understanding and promoting the acceptance of urinary health technologies among both patients and health care professionals.

## Methods

### Study Design

A cross-sectional study was conducted using a structured survey questionnaire developed to collect data from a variety of health care professionals, including nurses, physicians, nurse practitioners, and other patient-facing professionals. The survey included both closed- and open-ended questions to capture a wide range of perspectives and experiences. Pilot testing was conducted with a small group of health care professionals to refine the survey’s clarity, relevance, and ease of use. The final survey instrument covered a range of topics, including health care professionals’ experiences with urinary issues, their attitudes toward various technologies for urinary management, perceived barriers to and facilitators of technology adoption, and demographic information. By administering this carefully crafted survey, we sought to obtain valuable insights into the current landscape of urinary care and the potential for technological interventions to improve patient outcomes and health care efficiency.

### Recruitment

For this study, participants were recruited through a combination of social media platforms, specifically Reddit, and personal networks. The recruitment process aimed to gather a diverse sample representative of the target population. This form of recruitment could introduce bias into the sample and has certain limitations that will be described in greater detail in the *Limitations* section of this paper.

Reddit, a widely used online forum and community platform, was used for recruitment to reach a broad and varied demographic. The research team identified relevant subreddits that aligned with the survey’s topic, ensuring a population with interests and experiences related to the study. Recruitment posts were created, which included a brief description of the survey’s purpose, eligibility criteria, estimated time commitment, and a link to the survey. To maximize visibility and engagement, posts were shared in multiple subreddits, and follow-up comments were provided to address questions and encourage participation. In some cases, moderators of specific subreddits were contacted in advance to ensure that the study met subreddit guidelines and was not perceived as spam.

In addition to Reddit, recruitment was extended through the research team’s personal networks, including professional and academic connections. Invitations were sent via email, direct messaging, and personal referrals to individuals who met the study’s eligibility criteria. These communications contained details of the survey, emphasizing confidentiality, voluntary participation, and potential benefits. To promote a snowball sampling effect, participants were encouraged to share the survey link with individuals in their networks who might be eligible to participate.

### Inclusion and Exclusion Criteria

There were 286 responses to the survey. After cleaning the data and applying the inclusion and exclusion criteria, we ended up with 256 participants included in the resultant study. Inclusion criteria were (1) current or previous employment as a health care provider in a patient-facing health care setting (list of professions included in survey), (2) age 18 years or older, (3) proficiency in English, and (4) completion of most of the survey. We chose to seek the perspectives of multiple health care providers because we wanted to figure out whether technology implementation and perception and acceptability of technology could be related to various professional perceptions that may be related to the job title or role. We also wanted to create a comprehensive assessment of technology integration needs from different potential users. A screening question at the beginning of the survey confirmed participants’ health care roles (eg, physician, nurse, allied health staff). Those who did not self-identify as health care professionals were screened out.

### Ethical Considerations

The study was conducted in compliance with ethical guidelines and was approved by the Institutional Review Board (IRB) of San Jose State University (IRB protocol #24-087). Prior to recruitment and data collection, all study procedures were reviewed and determined to meet the ethical standards for research involving human participants. All procedures involving human participants were conducted in accordance with ethical standards. Participants provided informed consent prior to participation, and confidentiality was maintained throughout the study.

### Data Collection

The survey was administered online using the Qualtrics survey platform, with an estimated completion time of 15 minutes per participant. Survey items were developed based on validated constructs from several surveys [19-21]. The instrument underwent expert review by clinicians and digital health researchers to ensure content validity. To support survey validation, the instrument was reviewed by a panel of 10 content experts in nursing, urology, and public health. Their feedback was used to refine question clarity, content relevance, and construct alignment, thereby enhancing content and face validity. Where appropriate, the survey drew upon existing validated instruments, such as the eHealth Literacy Scale (eHEALS) and others described in depth in the *Measurement* section [21,22]. A pilot test (n=10) was conducted to assess question clarity, item wording, and overall usability. The experts provided feedback on item relevance, wording, and alignment with theoretical constructs, supporting content and face validity. Based on their recommendations, several items were reworded for clarity, redundant questions were removed, and the response scale was refined. This process ensured that the instrument accurately captured the intended domains and was appropriate for the target population. Additionally, the pilot confirmed the feasibility and usability of the survey format, contributing to overall instrument validity prior to full-scale distribution.

### Data Analysis

Statistical analyses were conducted using IBM SPSS Statistics. Descriptive statistics were used to summarize participant demographics and key survey variables. Prior to inferential testing, we assessed the normality of continuous variables using the Shapiro-Wilk test and visual inspection of histograms and quintile-quintile (Q-Q) plots. Most key variables were normally distributed, with 1 major variable, health literacy, present as not normally distributed. Despite this deviation from normality, we used parametric tests, specifically Pearson correlation and multiple linear regression, based on central limit theorem (CLT), which supports the use of parametric methods when sample sizes are sufficiently large, typically over 200 participants (N=256). We also assessed regression assumptions, including linearity and multicollinearity. All variance inflation factor (VIF) values were below 2.0, indicating no significant multicollinearity between predictors.

Next, to examine associations between health care providers’ perceptions of technology. Pearson correlation analyses were conducted to examine the bivariate relationships between belief agreement and key independent variables. Additionally, simultaneous multiple regression was conducted to assess predictors of belief agreement, with independent variables and covariates. Cronbach α was computed to assess the internal reliability of survey subscales, with a threshold of .70 considered acceptable for reliability.

### Measurement

The survey questions were developed using validated constructs, TAM and the HBM, to assess factors influencing the acceptance of technology by health care professionals. HBM variables were assessed using a validated questionnaire adapted from Champion and Skinner’s study [19,20]. Several survey constructs were used to develop questions measuring the perceptions of innovation adoption, specifically looking at attributes of technology effectiveness and receptiveness of technology. Several specific Likert scale questions came from the Unified Theory of Acceptance and Use of Technology (UTAUT), as described by Venkatesh et al [20], and the Diffusion of Innovations framework by Rogers [21], which informed perceptions around innovation and adoption behaviors. To assess perceived usefulness and ease of use, which are central to technology adoption, items were adapted from TAM, originally developed by Davis [23]. Additionally, the survey incorporated elements from Shertzer’s work [24] on self-efficacy which addresses resistance to change. To measure digital literacy and comfort with online health tools, items were adapted from eHEALS developed by Norman and Skinner [25]. Usability perceptions of health IT systems were captured using items adapted from the Customizable Health IT Usability Evaluation Scale developed by Yen et al [26]. For clarity, we defined each of the variables in the statistical analysis (Table 1).

**Table 1:** Variable definitions and construct triangulation.

Variable	Definition
Belief agreement	Health care professionals’ agreement with the perception that technology is efficient and their confidence in its integration, patient acceptance, its transformative potential, and the value of investing in technological solutions for managing urinary issues
Health literacy	The extent to which health care professionals understand and apply information about urinary health and related technologies
Perceived burden	The level of difficulty or inconvenience health care professionals associate with adopting new urinary health and related technologies
Frequency	How often health care professionals encounter patients with urinary concerns or use urinary health and related technologies in practice
Bladder volume	The utility of the estimated or measured urine volume in the bladder, as assessed through various technologies
Device attributes	The specific features of urinary health and related technology that health care professionals consider important when evaluating its usefulness
Importance	The priority health care professionals assign to technology use in improving patient outcomes
Concern level	The degree of apprehension or hesitation health care professionals have regarding the adoption of urinary health and related technology
Patient receptiveness	The willingness and openness of patients to use urinary health and related technology as part of their health care routine
Integrating technology	The feasibility and ease with which health care professionals can incorporate technology into their workflow
Support services	The availability of technical assistance, training, and resources needed to facilitate the implementation of technology in clinical settings

## Results

### Reliability Analysis

To assess the internal consistency of the survey instrument, Cronbach α was calculated for the Likert scale questions. The overall survey instrument demonstrated strong internal consistency, with Cronbach α=.85, indicating good reliability. All individual constructs within the survey also showed good internal consistency, with Cronbach α values ranging from .80 to .90, supporting the reliability of the scale across multiple domains. This suggests that the survey items measuring health care professionals’ perceptions of urinary issues and technology adoption were highly consistent in capturing the intended constructs.

### Quantitative Results

Of the 256 respondents, 94 (36.7%) identified as male, 155 (60.5%) as female, 3 (1.2%) as nonbinary/third gender, and 4 (1.6%) as self-described or “preferred not to state.” The mean age was 36.37 (SD 8.39, range 22-75) years. The majority of respondents were physicians (n=171, 66.8%), registered nurses (n=42, 16.4%), and allied health staff (n=17, 6.6%). The demographic data can be seen in Table 2.

**Table 2:** Participant demographics (N=256).

Category			Value^a^
**Gender, n (%)**			
	Male	94 (36.7)	
	Female	155 (60.5)	
	Nonbinary/third gender	3 (1.2)	
	Self-described/prefer not to state	4 (1.6)	
**Occupation, n (%)**			
	Physician	171 (66.8)	
	Registered nurse	42 (16.4)	
	Allied health staff	17 (6.6)	
	Medical assistant	7 (2.7)	
	Medical technologist	6 (2.3)	
	Nurse practitioner	5 (2.0)	
	Licensed vocational nurse	2 (0.8)	
	Certified nurse assistant	1 (0.4)	
	Other	5 (2.0)	
**Age (years), mean (SD)**			36.37 (8.39)
	20-29	18 (7.0)	
	30-39	155 (60.5)	
	40-49	62 (24.2)	
	50-59	14 (5.5)	
	60-69	4 (1.6)	
	70-79	1 (0.4)	
	Other	2 (0.8)	
**Education, n (%)**			
	Master’s degree	97 (37.9)	
	Bachelor’s degree	93 (36.3)	
	Doctoral degree	26 (10.2)	
	Associate’s degree	17 (6.6)	
	Technical degree or working training	16 (6.2)	
	Other or prefer not to say	7 (2.7)	

^a^The sums of percentages might not add up to 100% due to rounding.

Pearson correlation analysis was conducted to examine the relationship between belief agreement and a range of independent variables related to health care providers’ perspectives on urinary care and technology integration (Table 3). All variables demonstrated statistically significant positive correlations with belief agreement (*P*<.001), indicating that as each factor increases, so does the level of agreement with beliefs about the value and utility of managing urinary issues with technology.

**Table 3:** Correlation between independent variables and belief agreement.

Variable	r	Mean (SD)	*P* value
Health literacy	0.591	29.285 (6.890)	<.001
Perceived burden	0.628	73.549 (18.546)	<.001
Frequency	0.497	46.714 (8.108)	<.001
Bladder volume (monitoring perceptions)	0.611	31.042 (5.520)	<.001
Device attributes	0.650	46.403 (9.514)	<.001
Importance (of technology)	0.572	38.436 (10.614)	<.001
Concern level	0.351	30.895 (5.046)	<.001
Patient receptiveness	0.612	32.615 (7.912)	<.001
Integrating technology	0.479	25.321 (4.393)	<.001
Support services	0.622	30.823 (5.697)	<.001

The strongest correlation was observed between device attributes (r=0.650, *P*<.001) and belief agreement, suggesting that perceptions of favorable device features are strongly associated with greater belief in the value of urinary health and related technology. Similarly, perceived burden (r=0.628, *P*<.001) and support services (r=0.622, *P*<.001) were also highly correlated with belief agreement, indicating that health care providers who perceive urinary conditions as burdensome and who value supportive resources tend to hold stronger beliefs in the benefit of technology-based solutions.

Notably, patient receptiveness (r=0.612, *P*<.001) and bladder volume–monitoring perceptions (r=0.611, *P*<.001) were also strongly correlated with belief agreement, highlighting the importance of both patient attitudes and clinical relevance in shaping provider beliefs. Health literacy (r=0.591, *P*<.001) and the importance of urinary management (r=0.572, *P*<.001) showed moderate-to-strong correlations, indicating that more informed health care providers and those who place higher importance on managing urinary issues are more likely to agree with positive beliefs about technology use.

Frequency (r=0.497, *P*<.001) and integration of technology into practice (r=0.479, *P*<.001) were moderately correlated with belief agreement, while the concern level (r=0.351, *P*<.001) had the weakest, yet still significant, correlation.

Multiple regression analysis (Table 4) showed the dependence of belief agreement on the various predictors. The perceived burden (β=.284, *P*=.01), device attributes (β=.371, *P*<.001), and integrating technology (β=.312, *P*<.001) were positively associated.

**Table 4:** Multiple regression model of belief agreement (N=256).

Variable^a^	B^b^ (SE)	β	t_237_ Test	*P* value
Health literacy	0.024 (0.046)	0.047	0.509	.61
Perceived burden	0.053 (0.020)	0.284	2.644	.01
Frequency	0.0 (0.034)	–0.001	–0.010	.99
Bladder volume	0.005 (0.052)	0.008	0.095	.92
Device attributes	0.13 (0.036)	0.371	3.573	<.001
Importance	–0.068 (0.035)	–0.216	–1.941	.05
Concern level	–0.038 (0.042)	–0.055	–0.899	.37
Patient receptiveness	0.053 (0.039)	0.121	1.362	.18
Integrating technology	0.236 (0.051)	0.312	4.579	<.001
Support services	0.044 (0.050)	0.076	0.888	.38
Female (vs male)	–0.181 (0.350)	–0.027	–0.516	.61
Registered nurse (vs physician)	0.328 (0.542)	0.037	0.605	.55
Licensed vocational nurse (vs physician)	1.182 (2.309)	0.024	0.512	.61
Medical assistant (vs physician)	0.857 (1.084)	0.038	0.791	.43
Nurse practitioner (vs physician)	–0.499 (1.655)	–0.014	–0.301	.76
Medical technologist (vs physician)	0.727 (0.992)	0.035	0.733	.46
Allied health staff (vs physician)	0.492 (0.662)	0.038	0.743	.46

^a^The reference group for gender is male and for occupation is physician.

^b^B: unstandardized regression coefficient.

## Discussion

### Principle Findings

The findings of this study highlight key factors influencing belief agreement regarding the implementation of technology. The bivariate analysis demonstrated strong correlations between belief agreement and multiple factors, including device attributes (r=0.650, *P*<.001), the perceived burden (r=0.628, *P*<.001), support services (r=0.622, *P*<.001 ), patient receptiveness (r=0.612, *P*<.001), bladder volume (r= 0.611, *P*<.001 ), health literacy (r= 0.591, *P*<.001), importance (r=0.572, *P*<.001), frequency (r=0.497, *P*<.001), integrating technology (r=0.479, *P*<.001), and the concern level (r=0.351, *P*<.001). Effect sizes, as interpreted using Cohen’s conventions, ranged from medium to large. These relationships suggest that both individual and systemic factors play significant roles in shaping attitudes toward integrating bladder-monitoring technologies.

The relationship with the perceived burden demonstrated that health care professionals who experience a higher burden when managing urinary issues tend to support technology adoption more strongly. This makes sense; if urinary care is seen as challenging and time-consuming, tools that could ease this burden (eg, wearable bladder sensors) are more appealing. The correlation between bladder volume and belief agreement likely stems from the perceived need for accurate, noninvasive bladder monitoring. Those who frequently assess bladder volume may see the value in advanced technology for continuous or more accessible measurement. The strongest correlation in the bivariate analysis was the relationship with device attributes. This means that the more health care professionals perceive the device as having useful and effective features (eg, ease of use, accuracy, integration with existing workflows), the more they agree that technology is beneficial for urinary care. This indicates that the attributes of the device play a crucial role in acceptance. Regarding patient receptiveness, this indicates that if health care professionals feel that patients will be receptive toward technology, they are more likely to accept it. Finally, support services are essential. The bivariate analysis suggested that without adequate support, health care professionals are less likely to accept technology and will have concerns about failures of technology. The strongest relationships were found with device attributes, the perceived burden, and support services, which means that the design and usability of the device, the urgency of addressing urinary care burdens, and the availability of technical assistance are key determinants in acceptance.

The results of this study highlight several key predictors influencing belief agreement regarding the integration of health care technology. Notably, the perceived burden, device attributes, and the integration of technology into practice were all positively associated with belief agreement, suggesting that when clinicians perceive technology as less burdensome, more functional, and better integrated into workflows, they are more likely to align with positive beliefs about its utility and relevance.

The strongest association was observed with device attributes (β=.371, *P*<.001), emphasizing the critical role of usability, reliability, and functionality in shaping clinician acceptance. This finding supports the existing literature suggesting that perceived usefulness and ease of use are foundational elements in technology adoption frameworks. When devices are perceived as practical and well designed, clinicians are more likely to view them as beneficial to patient care and professional practice. The integration of technology (β=.312, *P*<.001) was also a significant predictor, indicating that belief agreement is reinforced when technologies are seamlessly embedded in daily workflows rather than introduced as disruptive or isolated tools. This underscores the importance of implementation strategies that consider organizational readiness, change management, and workflow alignment in order to promote sustained adoption.

### Comparison With Prior Work

This study builds upon the existing literature on the adoption and integration of technology in health care, particularly in the context of urological care. Prior research has highlighted the potential of technology to improve patient outcomes, enhance efficiency, and reduce the burden on health care professionals. However, these studies have also identified significant barriers to technology adoption. Consistent with previous findings, this study confirms that the perceived burden is a significant concern among health care professionals [14]. Research has consistently shown that poorly designed or implemented technologies can lead to cognitive overload, disrupted workflows, and decreased job satisfaction [22]. These frameworks emphasize the importance of perceived benefits, barriers, and self-efficacy in determining technology adoption. Prior studies have emphasized the critical role of training and support in facilitating technology integration. Effective training programs can enhance self-efficacy, minimize resistance to change, and improve the overall user experience [22,27]. This study reinforces these findings, highlighting the importance of integrating technology into existing systems and providing ongoing technical support. The positive association between device attributes and belief agreement is consistent with the research on technology acceptance. A user-friendly design, ease of use, and perceived usefulness are key factors that influence health care professionals’ willingness to adopt new technologies [28]. This study acknowledges that resistance to change and lack of integration into workflows can inhibit technology integration. Prior work has explored strategies for overcoming resistance, such as involving end users in the design process, providing clear communication about the benefits of technology, and addressing concerns about job security [29]. Prior studies have also shown the impact of early detection and trend evaluation using technologies such as tomography, which have increased the detection of symptomatic stones. This study supports the continued use of advanced technologies to improve detection and contribute to a better understanding of the increased incidence of kidney stones.

### Strengths

This study builds upon previous research by specifically examining health care professionals’ perceptions of urinary issues. This focused approach enables a deeper understanding of the unique challenges and opportunities within this area of health care relating to implementation of technologies. By integrating both quantitative and qualitative data, the study offers a comprehensive perspective on the factors influencing technology adoption. The qualitative analysis provides valuable insights into health care professionals’ experiences and viewpoints, complementing the statistical trends identified in the quantitative findings. These findings offer crucial guidance for clinicians, developers, and policymakers aiming to facilitate the adoption of these innovations.

### Limitations

Although this study provides valuable insights into health care professionals’ perceptions of urinary issues and the acceptability of technology for managing them, it is essential to acknowledge its limitations. There is a potential for sample bias. Participants were recruited through social media platforms (Reddit) and personal networks, which may introduce selection bias. This method might overrepresent individuals who are more tech savvy or active online or other personal factors, potentially skewing the results. The majority of respondents were physicians (66.8%), which limits the representation of other health care professionals, such as nurses and allied health staff, whose perceptions may differ.

Data were collected through a structured survey questionnaire, relying on self-reported perceptions and experiences. This method is subject to recall bias and social desirability bias, as participants may provide responses they believe are more acceptable or desirable. The study used a cross-sectional design, which captures data at a single point in time. This design limits the ability to establish causality or understand how perceptions and attitudes change over time. Longitudinal studies would be beneficial to track the evolution of technology acceptance and its impact on clinical practice. Finally, multiple regression analysis revealed several correlations between independent variables and belief agreement. However, correlation does not equal causation, and there may be other confounding variables not accounted for in the model.

### Recommendations

To support the effective adoption of urinary health technologies, 3 key strategies emerge from this study. First, user-centered design must be prioritized. Technologies should be intuitive, reliable, and seamlessly integrated into existing clinical workflows to enhance usability and encourage clinician engagement. When devices are perceived as practical and easy to use, they are more likely to be adopted and sustained in practice.

Structured training and ongoing support were cited as critical by respondents, particularly for technologies that involve patient monitoring or data interpretation. Ensuring that clinical staff receive role-relevant training and have access to timely assistance may reduce resistance and increase confidence in use. Some strategies to bolster support are to establish comprehensive training and a robust support infrastructure. Initial onboarding, ongoing education, and easily accessible technical assistance are critical to building confidence among health care professionals and reducing resistance to new tools. These efforts can also promote consistent and effective use of technology over time.

Finally, implementation strategies must address the perceived burden of technology use by aligning new tools with existing routines. Technologies that streamline tasks, reduce duplication, and enhance efficiency without increasing the provider burden are more likely to gain clinician acceptance and improve patient care outcomes. Aligning new tools with real-world clinical demands is essential to improving uptake in this specialized area.

### Conclusion

This study provides important insights into health care professionals’ perceptions of urinary care and the acceptability of wearable and remote technologies to manage such urinary conditions. Our findings highlight that belief agreement regarding the value of urinary health and related technology is significantly influenced by both individual perceptions and system-level factors. Specifically, the strongest correlates of belief agreement include device attributes, the perceived burden, and support services, with device usability, design, and functionality emerging as central to clinician acceptance. Notably, the perceived burden, device attributes, and integration of technology into workflows were also significant predictors of belief agreement in the regression model, emphasizing that practical implementation concerns remain critical for successful adoption.

Although other variables, such as health literacy, bladder volume, and patient receptiveness, were strongly correlated with belief agreement, they did not independently predict belief agreement in the multivariate model. This suggests that although these factors are influential, the most immediate drivers of acceptance are those tied to usability, burden relief, and ease of implementation.

Importantly, the study affirms that health care professionals are more likely to support the use of technology in urologic care when it is perceived as a meaningful solution to existing clinical burdens and when they feel confident in its practical integration. These findings align with prior literature and underscore the need for user-centered design, workflow-sensitive implementation strategies, and ongoing technical support and training.

The several limitations of this study, including the use of Reddit and personal networks for participant recruitment, may introduce selection bias, potentially overrepresenting those more comfortable with technology or engaged online. Additionally, the cross-sectional and self-reported nature of the data limits causal inference and may be subject to recall and social desirability bias. Future research should adopt longitudinal designs and include more diverse professional perspectives, including nurses and allied health staff, to better understand evolving attitudes over time.

Overall, this study offers actionable insights for developers, educators, and health system leaders seeking to promote the thoughtful integration of urinary health technologies that could be further explored in depth. Addressing the perceived burden, prioritizing user-friendly designs, and embedding technology into existing workflows will be critical for maximizing uptake and improving outcomes in urinary care.

## Abbreviations

AI: artificial Intelligence
eHEALS: eHealth Literacy Scale
EHR: electronic health record
HBM: Health Belief Model
IRB: Institutional Review Board
TAM: Technology Acceptance Model
UI: urinary incontinence
UTI: urinary tract infection
